# Supplementary material for: Catalytic activity tunable ceria nanoparticles prevent chemotherapy-induced acute kidney injury without interference with chemotherapeutics
Source: Nat Commun. 2021 Mar 4;12:1436. doi: 10.1038/s41467-021-21714-2 (PMC7933428; doi:10.1038/s41467-021-21714-2)
Supplement: Supplementary file 1 — Supplementary Information [file 41467_2021_21714_MOESM1_ESM.pdf]

Catalytic activity tunable ceria nanoparticles prevent chemotherapy-induced acute kidney injury without interference with chemotherapeutics

Weng *et al*.

Supplementary Figures

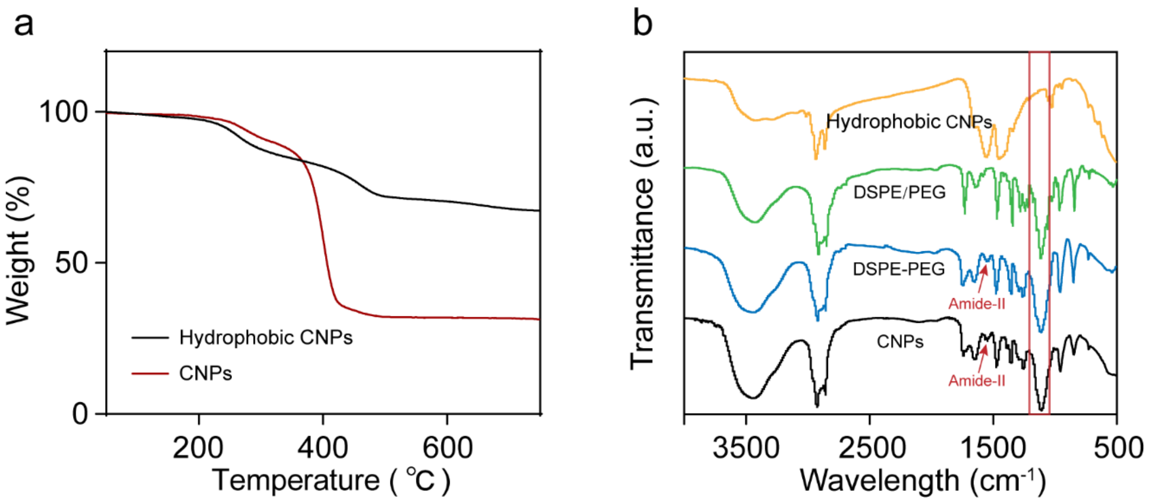

Supplementary Figure 1. Characterization of DSPE-PEG modification on CNPs. a, b, TGA curves of hydrophobic CNPs and CNPs (**a**) and Fourier transform infrared (FT-IR) spectra of hydrophobic CNPs, physical mixture of DSPE and PEG-COOH (DSPE/PEG), DSPE-PEG and CNPs (**b**). Source data are provided as a Source Data file.

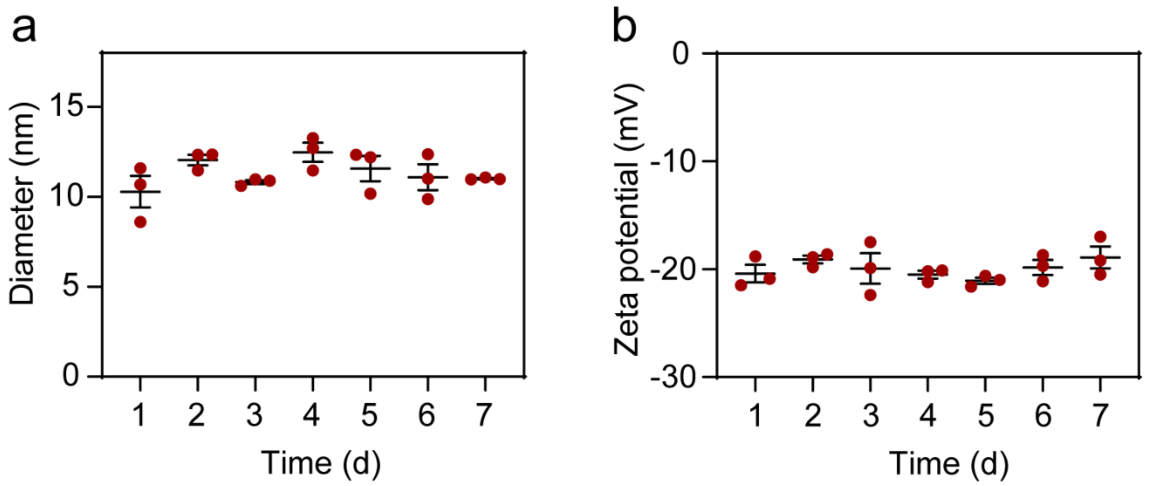

Supplementary Figure 2. Characterization of the sizes and zeta potentials of CNPs. a, b, The sizes (**a**) and zeta potentials (**b**) of CNPs over a week. Data are presented as mean values ± SEM. (n = 3 independent experiments). Source data are provided as a Source Data file.

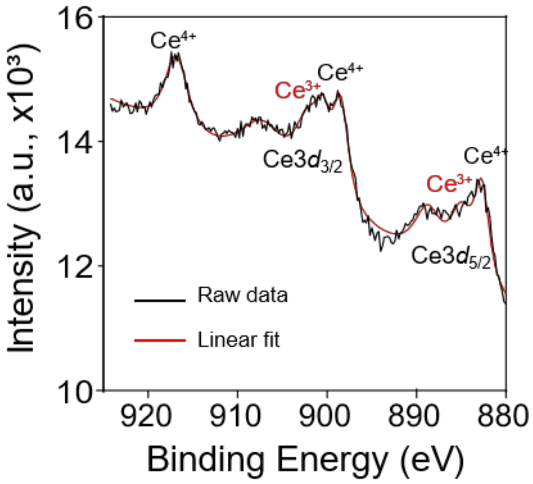

Supplementary Figure 3. X-ray photoelectron spectroscopy (XPS) analysis of CNPs. XPS spectra of CNPs shows corresponding binding energy peak of Ce (III) (885.0 and 903.5 eV) and Ce (IV) (882.1, 888.1, 898.0, 900.9, 906.4, and 916.4 eV). Source data are provided as a Source Data file.

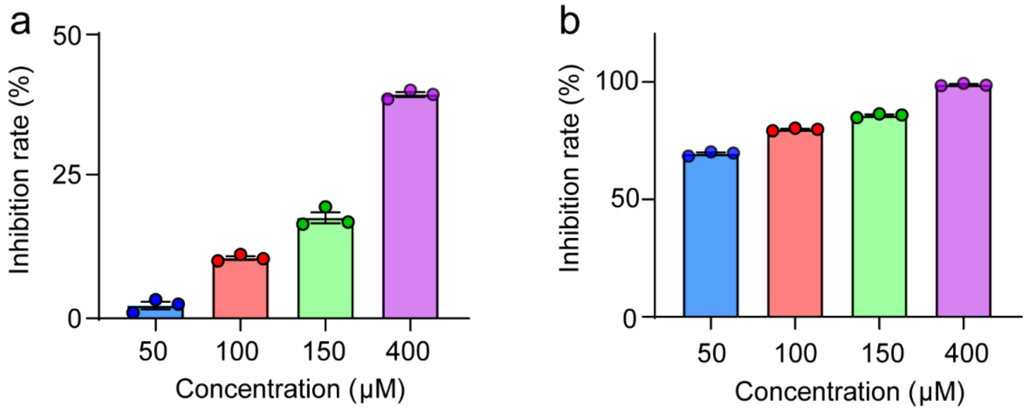

Supplementary Figure 4. Superoxide dismutase (SOD)-like activity of CNPs. a, b, SOD-like activity of CNPs at pH 7.4 (**a**) and pH 6.6 (**b**). Data are presented as mean values ± SEM. (n = 3 independent experiments). Source data are provided as a Source Data file.

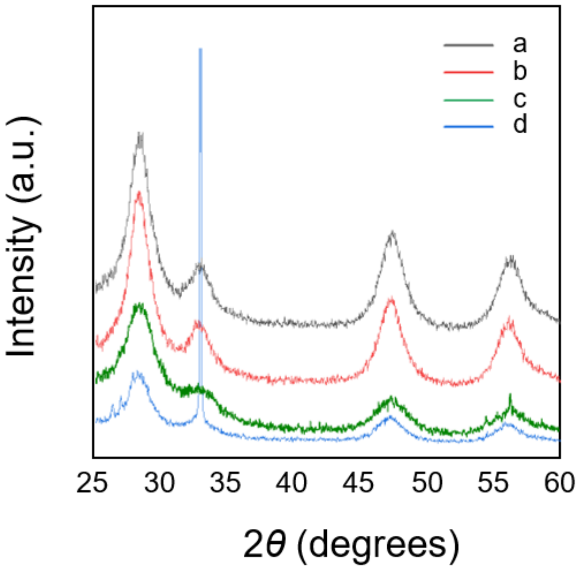

Supplementary Figure 5. X-ray powder diffraction (XRD) patterns of CNPs after different treatments. a, Untreated CNPs; **b**, The CNPs treated with H_2O_2 at pH 7.4 for 5 min; **c**, The CNPs treated with H_2O_2 at pH 6.6 for 5 min; **d**, The CNPs treated with H_2O_2 at pH 6.0 for 5 min. Source data are provided as a Source Data file.

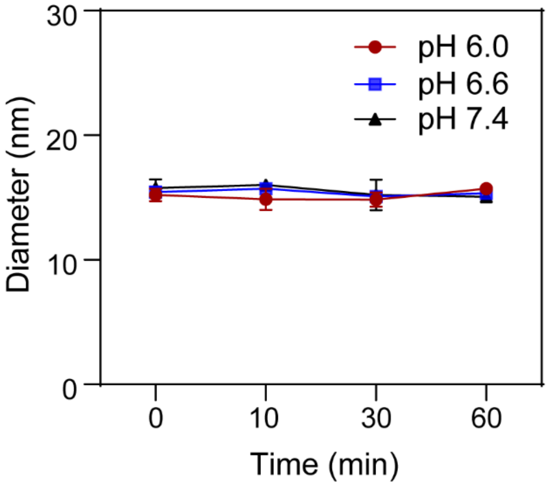

Supplementary Figure 6. Measurement of the colloidal stability of CNPs in the presence of H_2O_2. The hydrodynamic size of CNPs in the presence of H_2O_2 at pH 6.0, pH 6.6 and pH 7.4 at different time points (0, 10, 30, 60 min). Data are presented as mean values ± SEM. (n = 3 independent experiments). Source data are provided as a Source Data file.

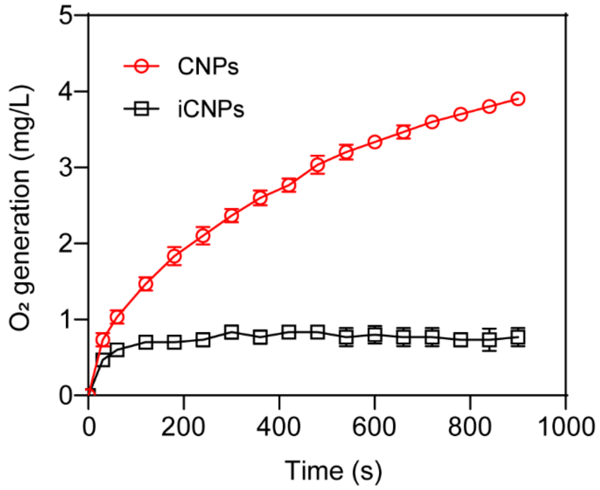

Supplementary Figure 7. The catalase-like activities of CNPs and inactive CNPs (iCNPs, H_2O_2 and H^+ pretreated CNPs). The catalase-like activities of CNPs and iCNPs under neutral condition (pH 7.4). Data are presented as mean values ± SEM. (n = 3 independent experiments). Source data are provided as a Source Data file.

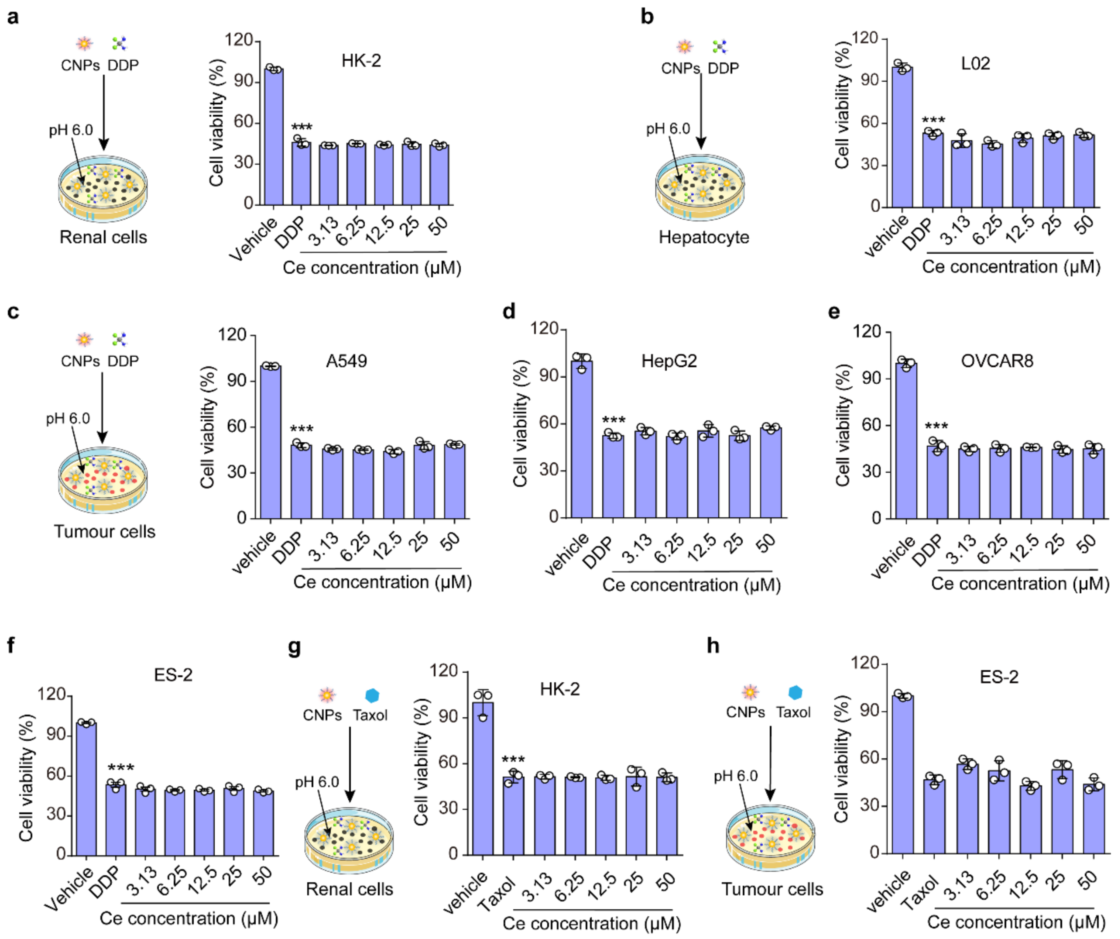

Supplementary Figure 8. CNPs display no cytoprotective effects at pH 6.0. a, The survival rate of HK-2 cells upon treatments with 10 μM cisplatin (DDP) and different concentrations of CNPs at pH 6.0. n = 3 independent experiments, $P_{(DDP)}$ = 3.8E-14. **b**, The survival rate of L02 cells upon treatments with 10 μM DDP and different concentrations of CNPs at pH 6.0. n = 3 independent experiments, $P_{(DDP)}$ = 3.6E-10. **c-f**, The survival rate of A549 cells (**c**), HepG2 cells (**d**), OVCAR8 cells (**e**) and ES-2 cells (**f**) upon treatments with 10 μM DDP and different concentrations of CNPs at pH 6.0. n = 3 independent experiments. In **c**, $P_{(DDP)}$ = 3.8E-14, in **d**, $P_{(DDP)}$ = 2.8E-10, in **e**, $P_{(DDP)}$ = 6E-12, in **f**, $P_{(DDP)}$ = 7.24E-13. **g**, The survival rate of HK-2 cells upon treatments with 5 μM Taxol and different concentrations of CNPs at pH 6.0. n = 3 independent experiments, $P_{(DDP)}$ = 3.8E-08. **h**, The survival rate of ES-2 cells upon treatments with 5 μM Taxol and different concentrations of CNPs at pH 6.0. n = 3 independent experiments, $P_{(DDP)}$ = 4.6E-09. Data are presented as means ± SEM., ****P* < 0.001 compared to vehicle; one-way ANOVA with multiple comparisons test. Source data are provided as a Source Data file.

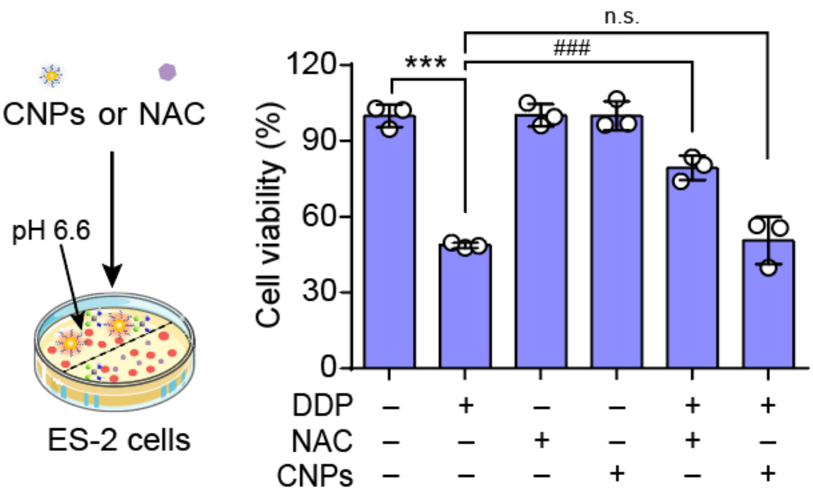

Supplementary Figure 9. The cytoprotective effects of CNPs and N-acetyl cysteine (NAC). The survival rate of ES-2 cells after different treatments at pH 6.6. n = 3 independent experiments, $P_{(DDP)}$ = 1.1E-06, $P_{(DDP+NAC)}$ = 0.00023, $P_{(DDP+CNPs)}$ = 0.9975. Data are presented as means ± SEM., ****P* < 0.001, ###*P* < 0.001, n.s., no significance; one-way ANOVA with multiple comparisons test. Source data are provided as a Source Data file.

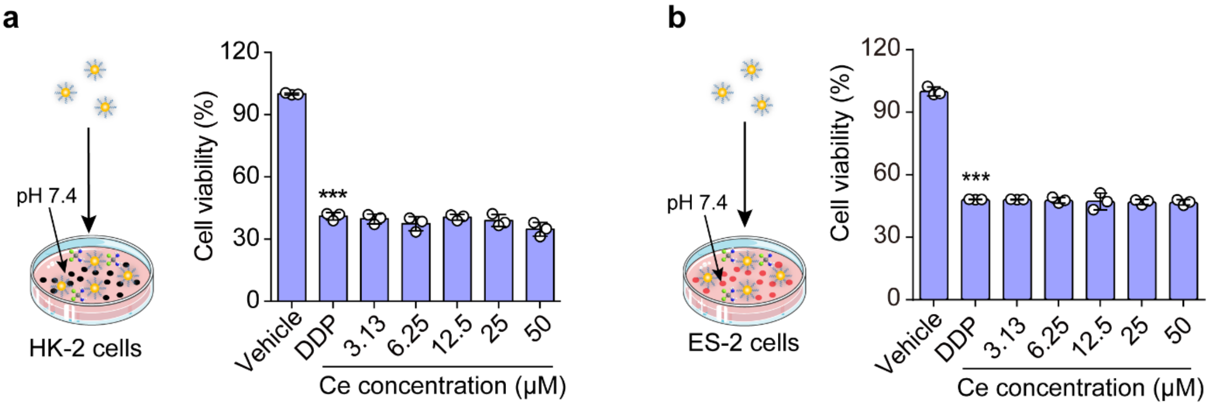

Supplementary Figure 10. a, b, The cytoprotective effects of iCNPs at pH 7.4. The survival rate of HK-2 cells (**a**) and ES-2 cells (**b**) after treatments with 10 μM DDP and different concentrations of iCNPs at pH 7.4. The iCNPs were obtained by pre-treatment with H_2O_2 under acidic condition. n = 3 independent experiments. In **a**, $P_{(DDP)}$ = 4E-13; in **b**, $P_{(DDP)}$ = 1.1E-13. Data are presented as means ± SEM., ****P* < 0.001 compared to vehicle; one-way ANOVA with multiple comparisons test, Source data are provided as a Source Data file.

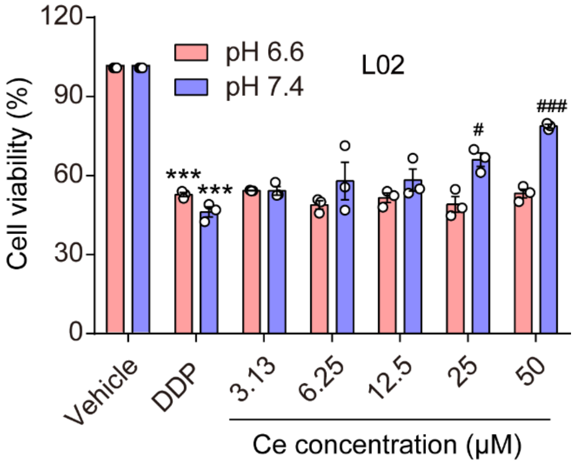

Supplementary Figure 11. The protective effects of CNPs on hepatocytes. The survival rate of hepatocytes (L02) after treatments with 10 μM DDP and different concentrations of CNPs at pH 7.4 and pH 6.6. n = 3 independent experiments, $P_{(DDP, 6.6)}$ = 4.4E-10, $P_{(DDP, 7.4)}$ = 4.6E-07, $P_{(25, 7.4)}$ = 0.015, $P_{(50, 7.4)}$ = 0.00017. Data are presented as means ± SEM., ***$P < 0.001$ compared to vehicle, #$P < 0.05$, ###$P < 0.001$ compared to DDP; one-way ANOVA with multiple comparisons test. Source data are provided as a Source Data file.

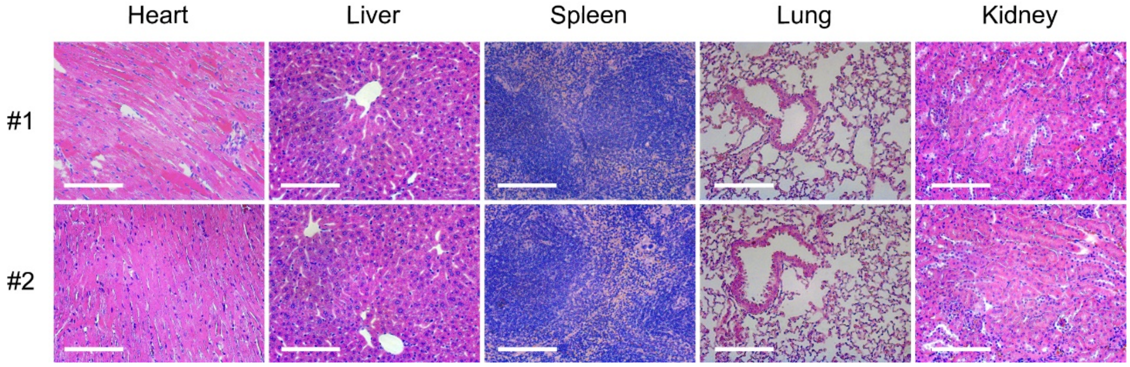

Supplementary Figure 12. The hematoxylin and eosin-stained sections of major organs in CNP-treated mice. Representative hematoxylin and eosin-stained sections of heart, liver, spleen, lung and kidney in two mice treated with 1.5 mg/kg CNPs alone after 72 h. n = 2 independent animals. Scale bars: 200 μm.

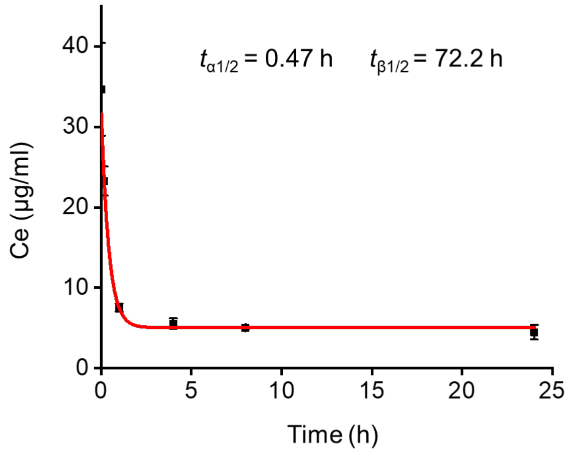

Supplementary Figure 13. In vivo pharmacokinetic curves of CNPs. The blood circulation profiles of CNPs fitted well with the classic two-compartment pharmacokinetic model, and the terminal elimination half-lives of the central component and peripheral component were calculated to be 0.47 and 72.2 h, respectively. n = 3 independent animals. Source data are provided as a Source Data file.

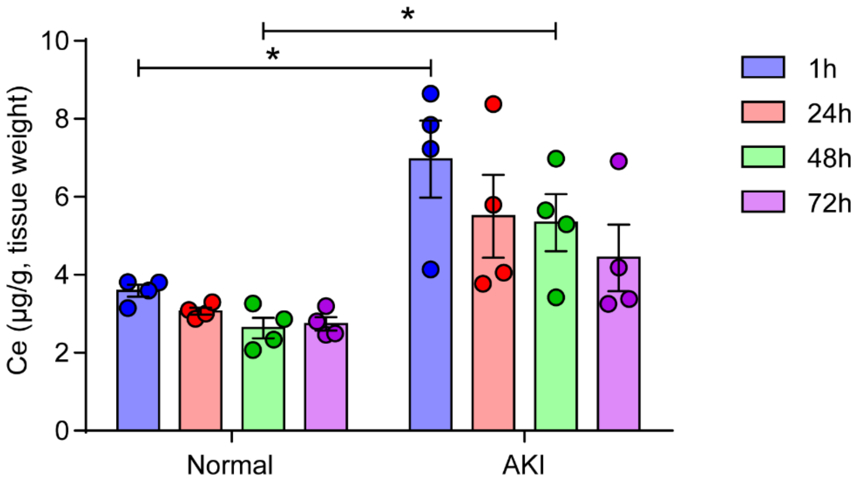

Supplementary Figure 14. The accumulation of CNPs in the kidneys of normal and AKI mice. Cerium ion levels in the kidneys of healthy mice and AKI mice at different time points after intravenous administration of CNPs (1.5 mg/kg). n = 4 independent experiments, $P_{(1h)}$ = 0.0148, $P_{(48h)}$ = 0.0135. Data are presented as means ± SEM., *P < 0.05; two-tailed unpaired Student's t-test. Source data are provided as a Source Data file.

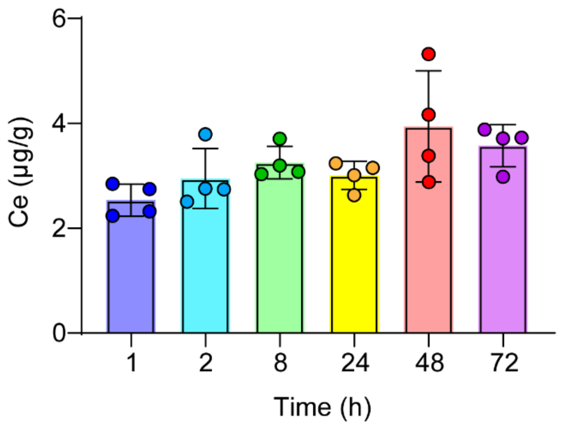

Supplementary Figure 15. The accumulation of CNPs in the renal cortex of AKI mice. Cerium ion levels in renal cortex of AKI mice at different time points (1, 2, 8, 24, 48, 72 h) after intravenous administration of CNPs (1.5 mg/kg). n = 4 independent renal cortex tissues at each time point. Source data are provided as a Source Data file.

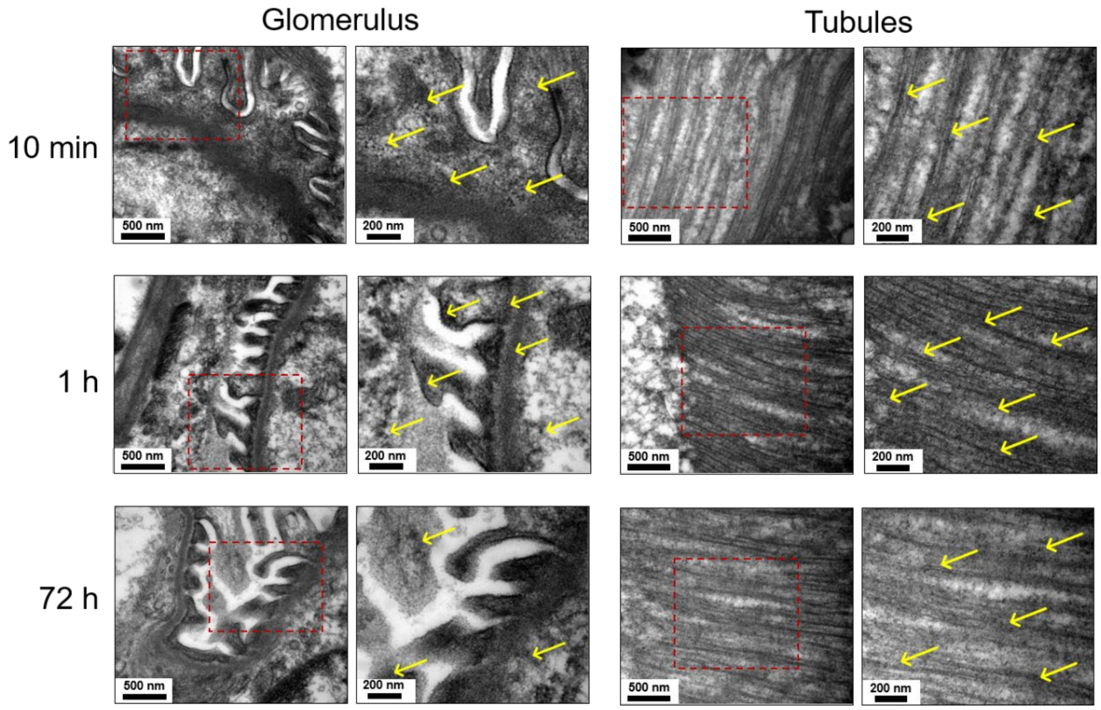

Supplementary Figure 16. Biodistribution of CNPs in the renal cortex of AKI mice. The TEM images of CNPs in the renal cortex of AKI mice at different time points (10 min, 1 h, 72 h) after intravenous administration of CNPs (1.5 mg/kg). Yellow arrows indicate the presence of CNPs. n = 3 independent renal cortex tissues at each time point. Source data are provided as a Source Data file.

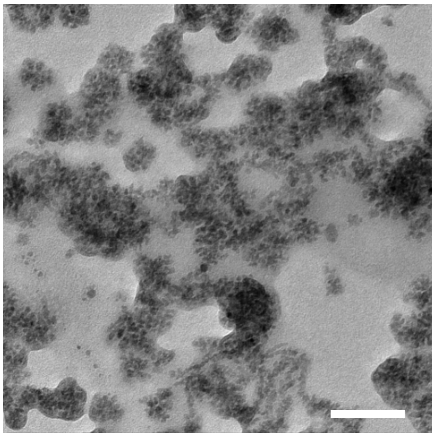

Supplementary Figure 17. The renal excretion of CNPs in AKI mice. TEM image of the urine collected from AKI mice at 2 h after intravenous administration of CNPs (1.5 mg/kg). Scale bar: 50 nm. n = 3 independent mice urine samples. Source data are provided as a Source Data file.

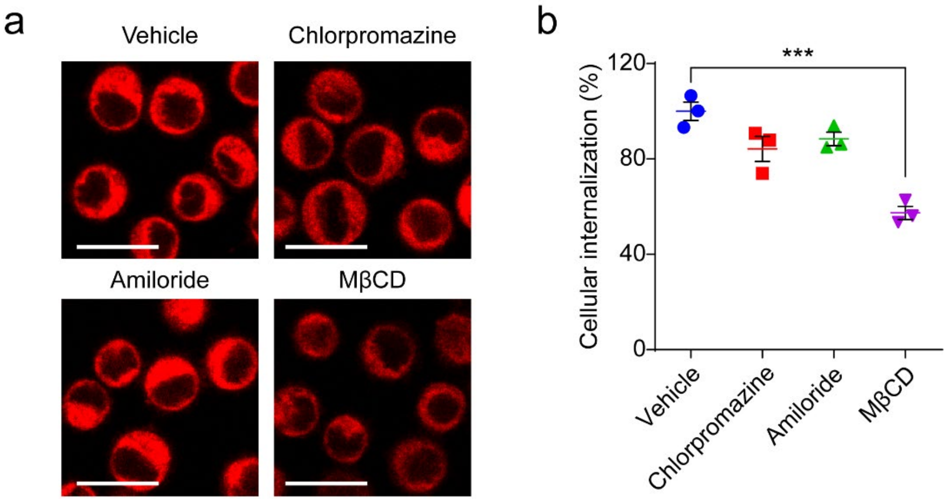

Supplementary Figure 18. The mechanism underlying the cellular endocytosis of CNPs in kidney cells. a, Confocal images of HK-2 cells pre-treated with serum-free medium (vehicle), chlorpromazine (an inhibitor of clathrin-mediated endocytosis), amiloride (an inhibitor of macropinocytosis) or methyl-b-cyclodextrin (MβCD, an inhibitor of caveolin-mediated endocytosis) followed by co-incubation with 50 μM CNPs. Scale bar: 20 μm. **b,** Quantitative analysis of fluorescence intensity of RITC-labeled CNPs in HK-2 cells after different pretreatments. n = 3 independent experiments, $P_{(M\beta CD)} = 0.000215$. Data are presented as means ± SEM., ***$P < 0.001$; one-way ANOVA with multiple comparisons test. Source data are provided as a Source Data file.

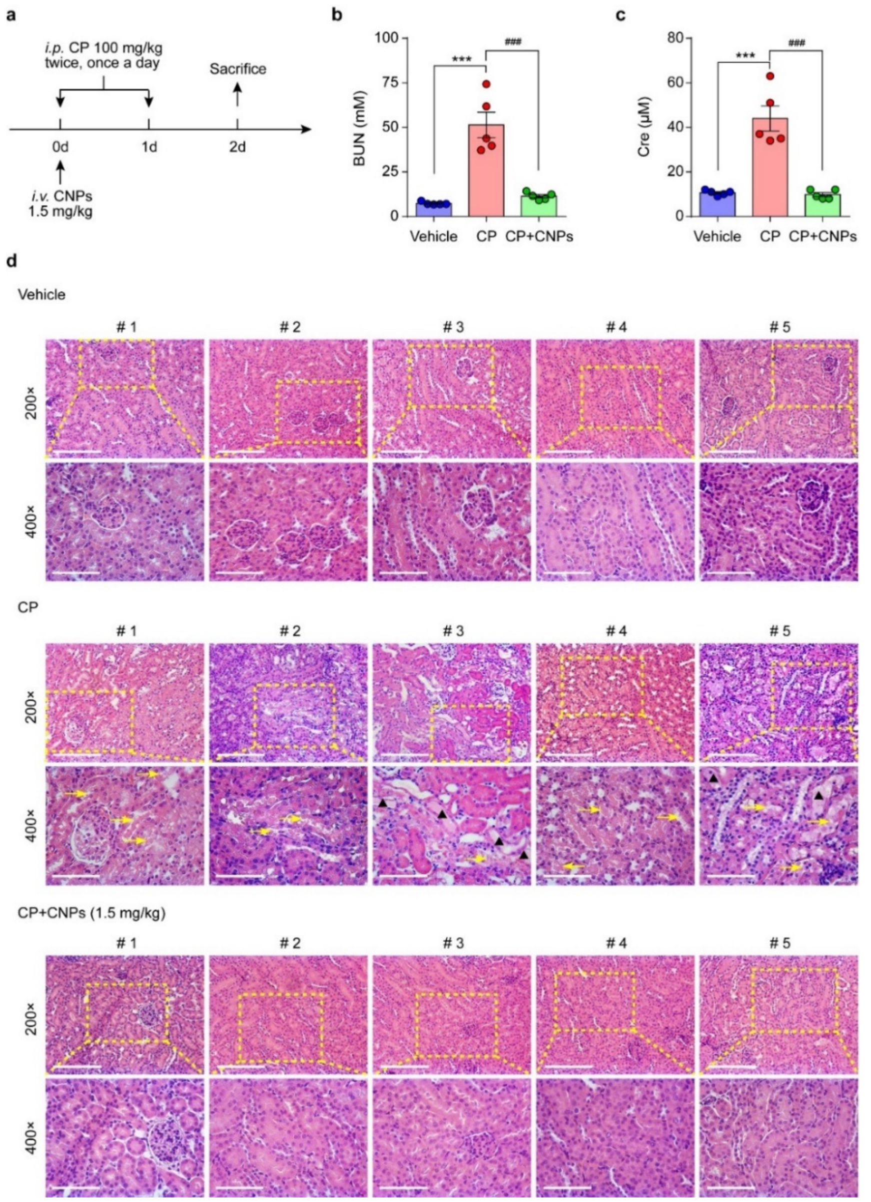

Supplementary Figure 19. CNPs protect against cyclophosphamide (CP)-induced AKI in vivo. a, Schematic representation of treatment schedule and therapy assessments for cyclophosphamide (CP)-induced AKI mice. **b**, **c**, Serum blood urea nitrogen (BUN) (**b**) and creatinine **(**Cre) (**c**) levels in each group. n = 5 independent animals. In **b**, $P_{(CP)}$ = 2.1E-5, $P_{(CP+CNPs)}$ = 5.6E-05; in **c**, $P_{(CP)}$ = 3.4E-05, $P_{(CP+CNPs)}$ = 2.7E-05. **d**, Representative H&E sections of the kidneys from each group. n = 5 independent animals. Arrows indicate tubules with necrosis, epithelial anoikis cavitation, or loss of brush border. Triangles denote the formation of casts in tubes. The lower panel are the magnified regions from the upper panel. Scale bar: 200 μm (up) or 100 μm (down). Data are presented as means ± SEM., ****P* < 0.001, ###*P* < 0.001; one-way ANOVA with multiple comparisons test. Source data are provided as a Source Data file.

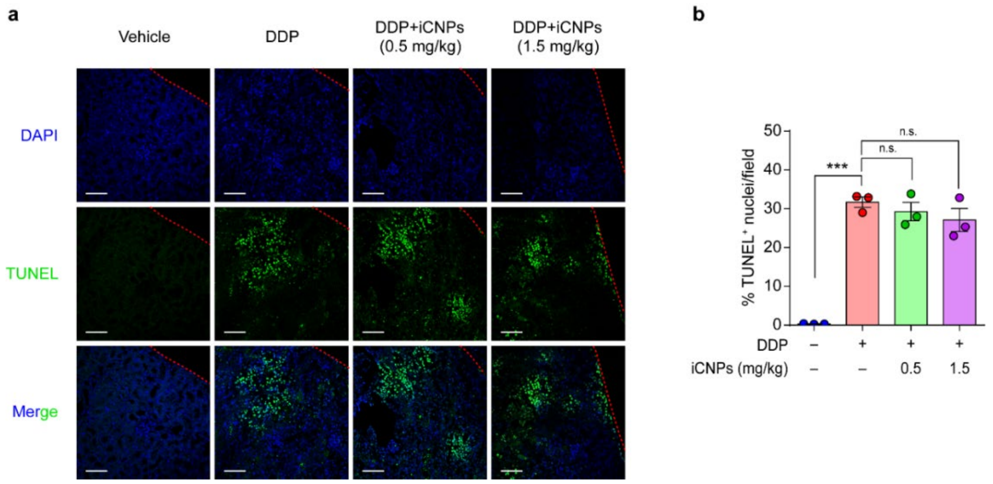

Supplementary Figure 20. iCNPs exert no protective effect on apoptosis in vivo. a, Representative TUNEL staining images in kidneys from mice treated with iCNPs. Scale bar: 100 μm. Red dotted line indicates the boundary between the kidney edge and background. n = 3 independent mouse kidneys. **b**, Quantification of TUNEL positive cells in the respective groups. n = 3 independent mouse kidneys, $P_{(DDP)}$ = 1.9E-05, $P_{(DDP+0.5CNPs)}$ = 0.8281, $P_{(DDP+1.5CNPs)}$= 0.4225. Data are presented as means ± SEM., ***P < 0.001, n.s., no significance; one-way ANOVA with multiple comparisons test. Source data are provided as a Source Data file.

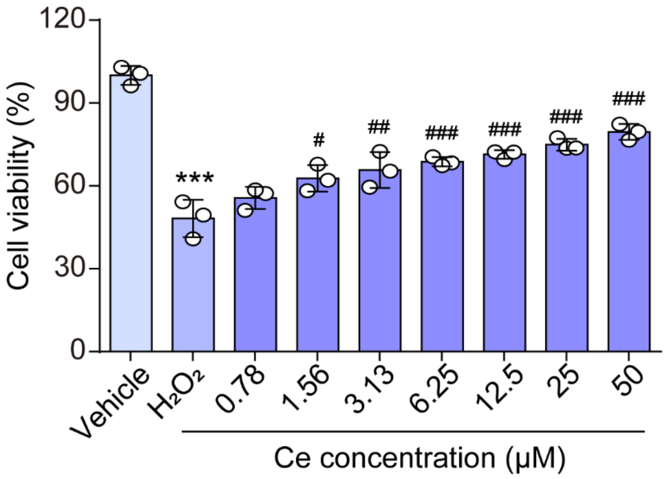

Supplementary Figure 21. CNPs protect against H_2O_2 in vitro. Cell viability of HK-2 cells treated with vehicle, 50 μM H_2O_2 and 50 μM H_2O_2 plus different concentrations of CNPs. n = 3 independent experiments, $P_{(H_2O_2)}$ = 3.2E-10, $P_{(1.56)}$ = 0.0108, $P_{(3.13)}$ = 0.0017, $P_{(6.25)}$ = 0.00029, $P_{(12.5)}$ = 6.2E-05, $P_{(25)}$ = 9.3E-06, $P_{(50)}$ = 9.4E-07. Data are presented as means ± SEM., ***P < 0.001 compared to vehicle, #P < 0.05, ##P < 0.01, ###P < 0.001 compared to H_2O_2; one-way ANOVA with multiple comparisons test. Source data are provided as a Source Data file.

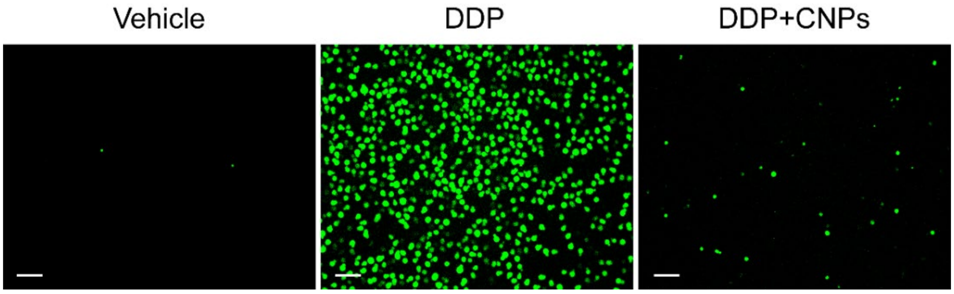

Supplementary Figure 22. The effects of CNPs to quench reactive oxygen species in vitro. Representative fluorescent images of HK-2 cells after the treatment with vehicle, DDP and DDP plus CNPs. Scale bar: 100 µm. n = 3 independent experiments.

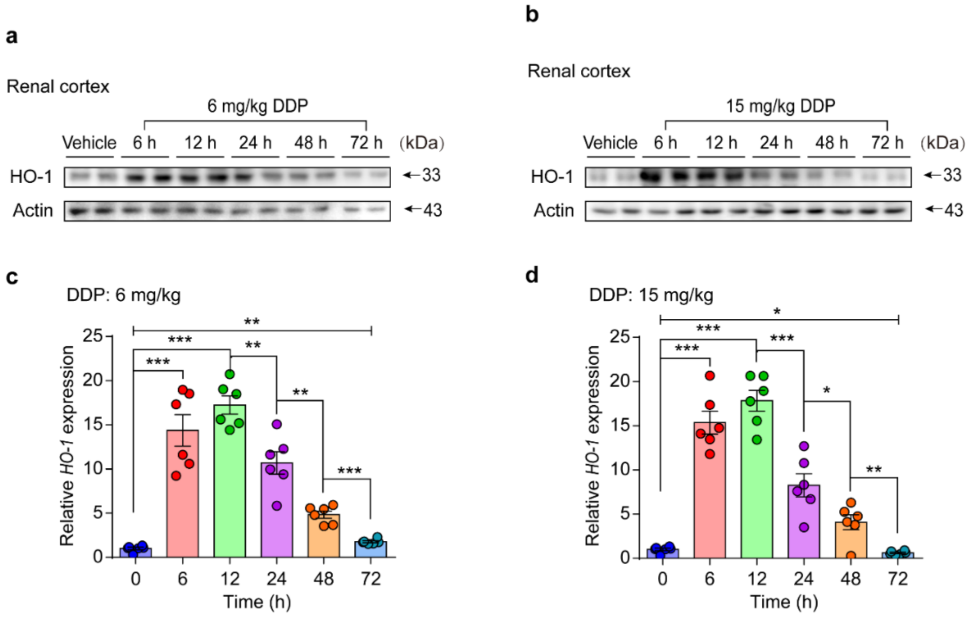

Supplementary Figure 23. The effects of CNPs on ROS-related protein and gene expression in vivo. a, **b**, The expression of HO-1 in renal cortex of mice after treatment with 6 mg/kg (**a**) and 15 mg/kg (**b**) DDP. Actin served as a loading control. n = 2 independent experiments. **c**, Relative mRNA expression of *HO-1* in renal cortex of mice after treatment with 6 mg/kg DDP. n = 3 independent experiments, $P_{(6\text{ h vs }0\text{ h})} = 2.1\text{E-}05$, $P_{(12\text{ h vs }0\text{ h})} = 2.3\text{E-}08$, $P_{(24\text{ h vs }12\text{ h})} = 0.0025$, $P_{(48\text{ h vs }24\text{ h})} = 0.0014$, $P_{(48\text{ h vs }72\text{ h})} = 3.3\text{E-}05$, $P_{(0\text{ h vs }72\text{ h})} = 0.0018$. **d**, Relative mRNA expression of *HO-1* in renal cortex of mice after treatment with 15 mg/kg DDP. n = 3 independent experiments, $P_{(6\text{ h vs }0\text{ h})} = 6.6\text{E-}07$, $P_{(12\text{ h vs }0\text{ h})} = 5.9\text{E-}08$, $P_{(24\text{ h vs }12\text{ h})} = 0.0003$, $P_{(48\text{ h vs }24\text{ h})} = 0.023$, $P_{(72\text{ h vs }48\text{ h})} = 0.0022$, $P_{(72\text{ h vs }0\text{h})} = 0.048$. Data are presented as means ± SEM., *$P < 0.05$, **$P < 0.01$, ***$P < 0.001$; statistical significance was calculated by two tailed Student's t-test. Source data are provided as a Source Data file.

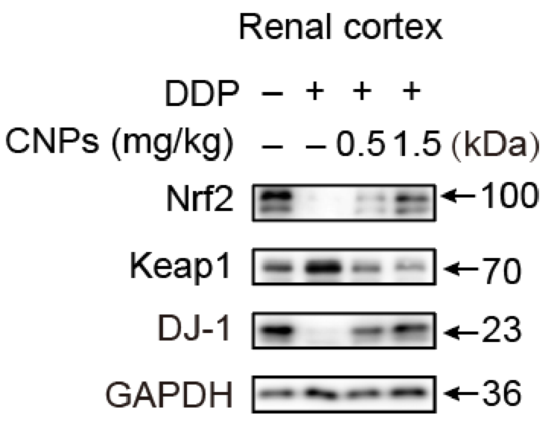

Supplementary Figure 24. The effects of CNPs on Nrf2, Keap1 and DJ-1 levels in vivo. Western blot analysis of Nrf2, Keap1 and DJ-1 levels in the renal cortex of mice treated with vehicle, DDP and DDP plus CNPs (0.5 mg/kg and 1.5 mg/kg). GAPDH served as a loading control. n = 2 independent experiments. Source data are provided as a Source Data file.

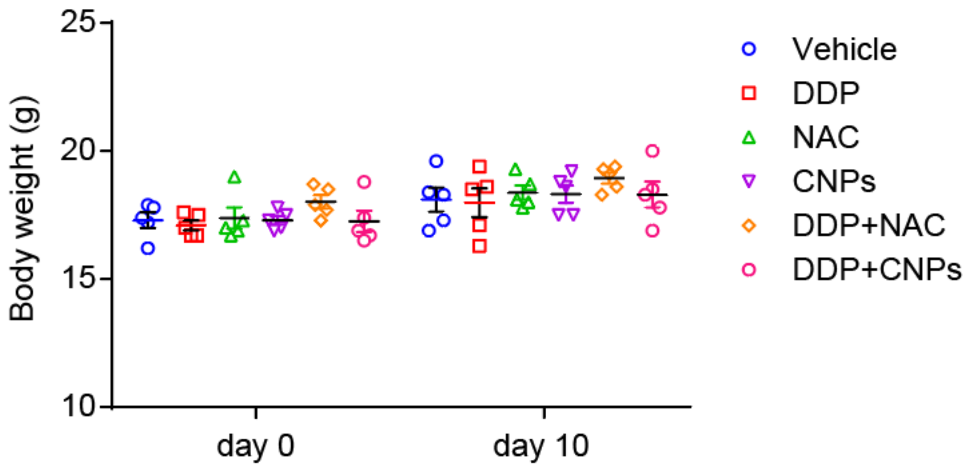

Supplementary Figure 25. The body weight changes of mice during the treatment. Body weight of tumour-bearing BALB/c nude mice fed with sterile water in day 0 and day 10 after treatment with DDP (intraperitoneal injection, 3 mg/kg, twice a week, total for twice), NAC (oral administration, 400 mg/kg, daily) and (or) CNPs (intravenous injection, 1.5 mg/kg, twice a week, total for twice). n = 5 independent animals, Data are presented as means ± SEM. Source data are provided as a Source Data file.

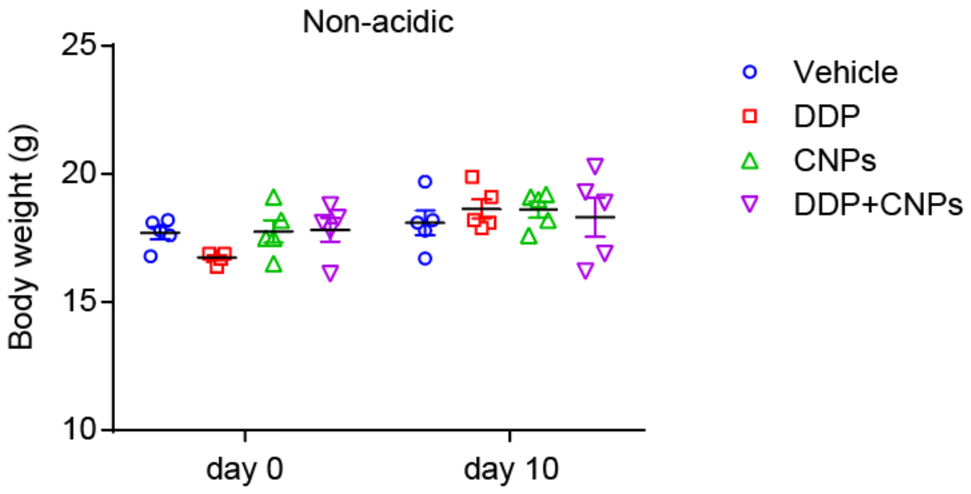

Supplementary Figure 26. The body weight changes of mice during the treatment. Body weight of tumour-bearing BALB/c nude mice fed with soda water in day 0 and day 10 after treatment with DDP (intraperitoneal injection, 3 mg/kg, twice a week, total for twice), NAC (oral administration, 400 mg/kg, daily) and (or) CNPs (intravenous injection, 1.5 mg/kg, twice a week, total for twice). n = 5 independent animals, Data are presented as means ± SEM. Source data are provided as a Source Data file.

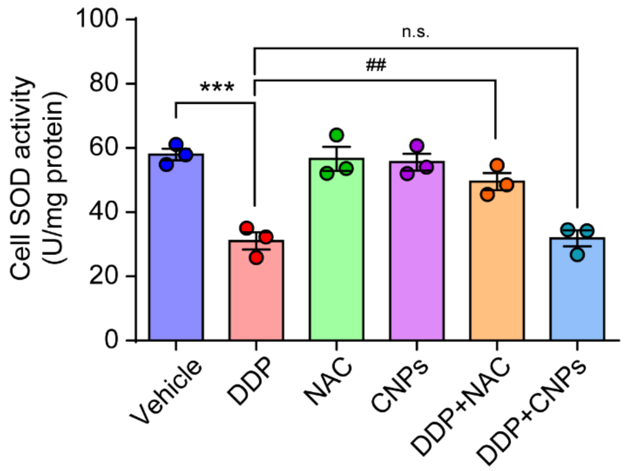

Supplementary Figure 27. The SOD activity in ES-2 cells after different treatments. SOD activity in ES-2 cells treated with DDP, NAC, CNPs, DDP+NAC and DDP+CNPs under acidic condition. n = 3 independent experiments, $P_{(DDP)} = 0.00017$, $P_{(DDP+NAC)} = 0.0046$, $P_{(DDP+CNPs)} = 1.000$. Data are presented as means ± SEM., $**P < 0.01$, $\#\#P < 0.01$, n.s., no significance; one-way ANOVA with multiple comparisons test. Source data are provided as a Source Data file.

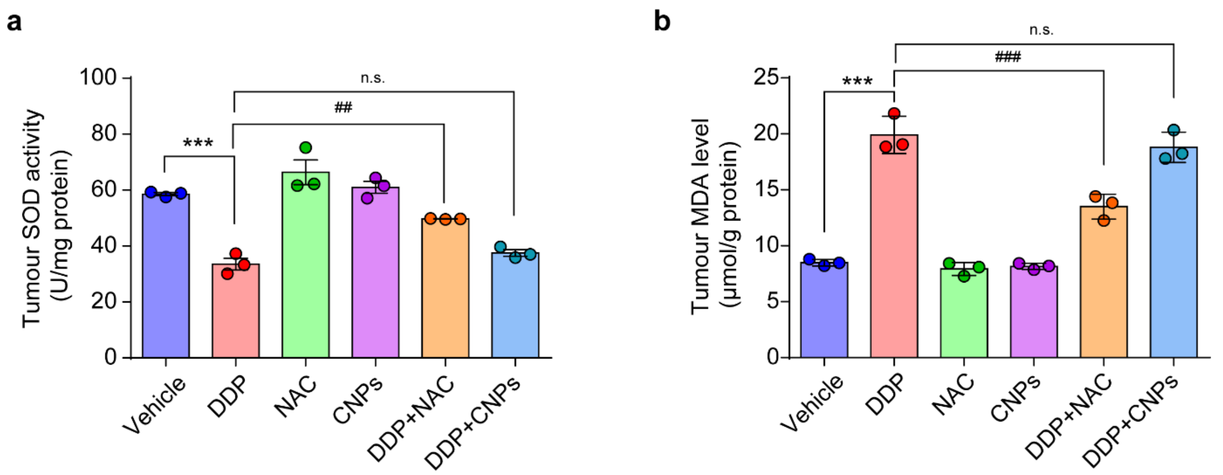

Supplementary Figure 28. The levels of oxidative stress of tumours after different treatments in vivo. The SOD activity (**a**) and MDA level (**b**) of tumour tissues from BALB/c mice fed with sterile water after different treatments. n = 3 independent experiments. In **a**, $P_{(DDP)}$ = 5E-05, $P_{(DDP+NAC)}$ = 0.0028, $P_{(DDP+CNPs)}$ = 0.8036; in **b**, $P_{(DDP)}$ = 1.4E-07, $P_{(DDP+NAC)}$ = 7E-05 , $P_{(DDP+CNPs)}$ = 0.7721. Data are presented as means ± SEM., ***P < 0.001, ##P < 0.01, ###P < 0.001, n.s., no significance; one-way ANOVA with multiple comparisons test. Source data are provided as a Source Data file.

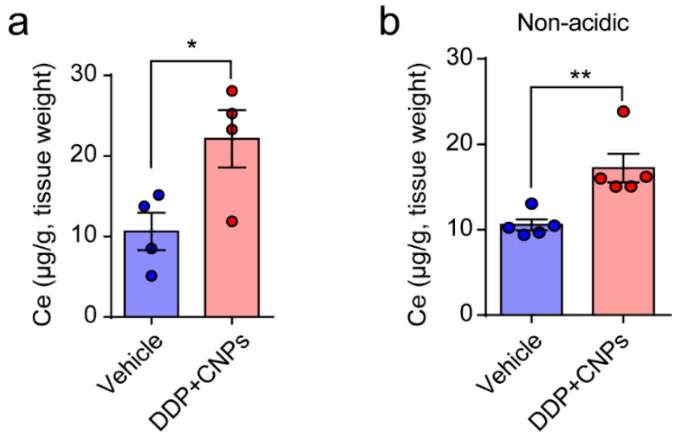

Supplementary Figure 29. The accumulation of CNPs in tumour tissues. a, Cerium ion levels in the tumours of nude mice fed with sterile water in the vehicle group and DDP+CNPs group. n = 4 independent mouse tumour tissues, P = 0.035. **b,** Cerium ion levels in the tumours of nude mice fed with sterile water in the vehicle group and DDP+CNPs group. n = 5 independent mouse tumour tissues, P = 0.0059. Data are presented as means ± SEM., *P < 0.05, **P < 0.01; statistical significance was calculated by two tailed Student's t-test. Source data are provided as a Source Data file.

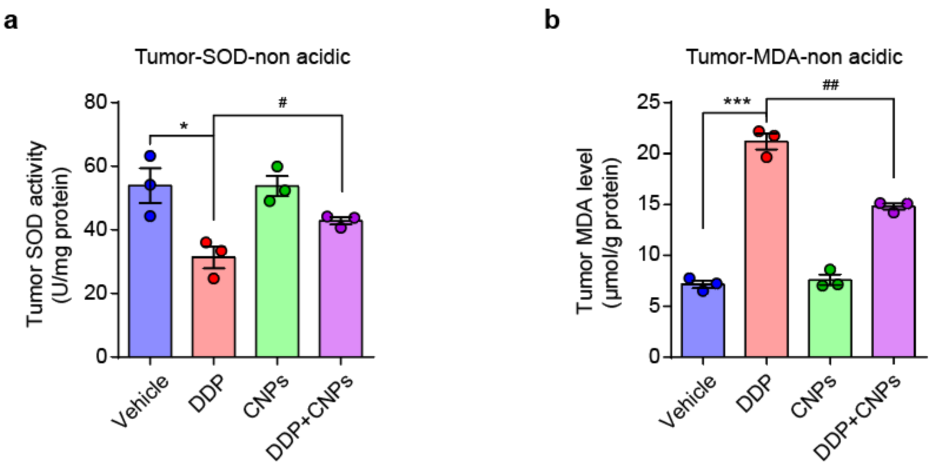

Supplementary Figure 30. The levels of oxidative stress of tumours after different treatments in vivo. The SOD activity (**a**) and MDA level (**b**) of tumour tissues from BALB/c mice fed with soda water after different treatments. n = 3 independent experiments. In **a**, $P_{(DDP)}$ = 0.025, $P_{(DDP+CNPs)}$ = 0.033; in **b**, $P_{(DDP)}$ = 9E-05, $P_{(DDP+CNPs)}$ = 0.00164. Data are presented as means ± SEM., *$P < 0.05$, ***$P < 0.001$; #$P < 0.05$, ##$P < 0.01$; statistical significance was calculated by two tailed Student's t-test. Source data are provided as a Source Data file.

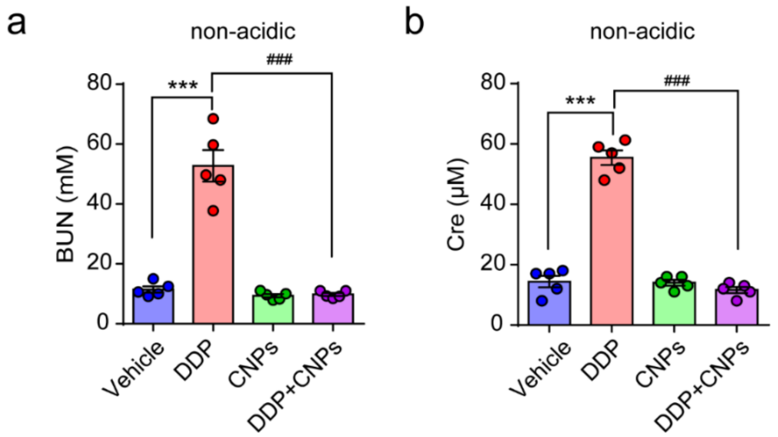

Supplementary Figure 31. The effects of CNPs on serum BUN and Cre levels. Serum BUN (**a**) and Cre (**b**) levels in nude mice fed with soda water after administration of DDP and (or) CNPs. n = 5 independent animals. In **a**, $P_{(DDP)}$ = 5.3E-08, $P_{(DDP+CNPs)}$ = 3E-08; in **b**, $P_{(DDP)}$ = 5.3E-11, $P_{(DDP+CNPs)}$ = 2E-11. Data are presented as means ± SEM., ***$P < 0.001$, ###$P < 0.001$; one-way ANOVA with multiple comparisons test. Source data are provided as a Source Data file.

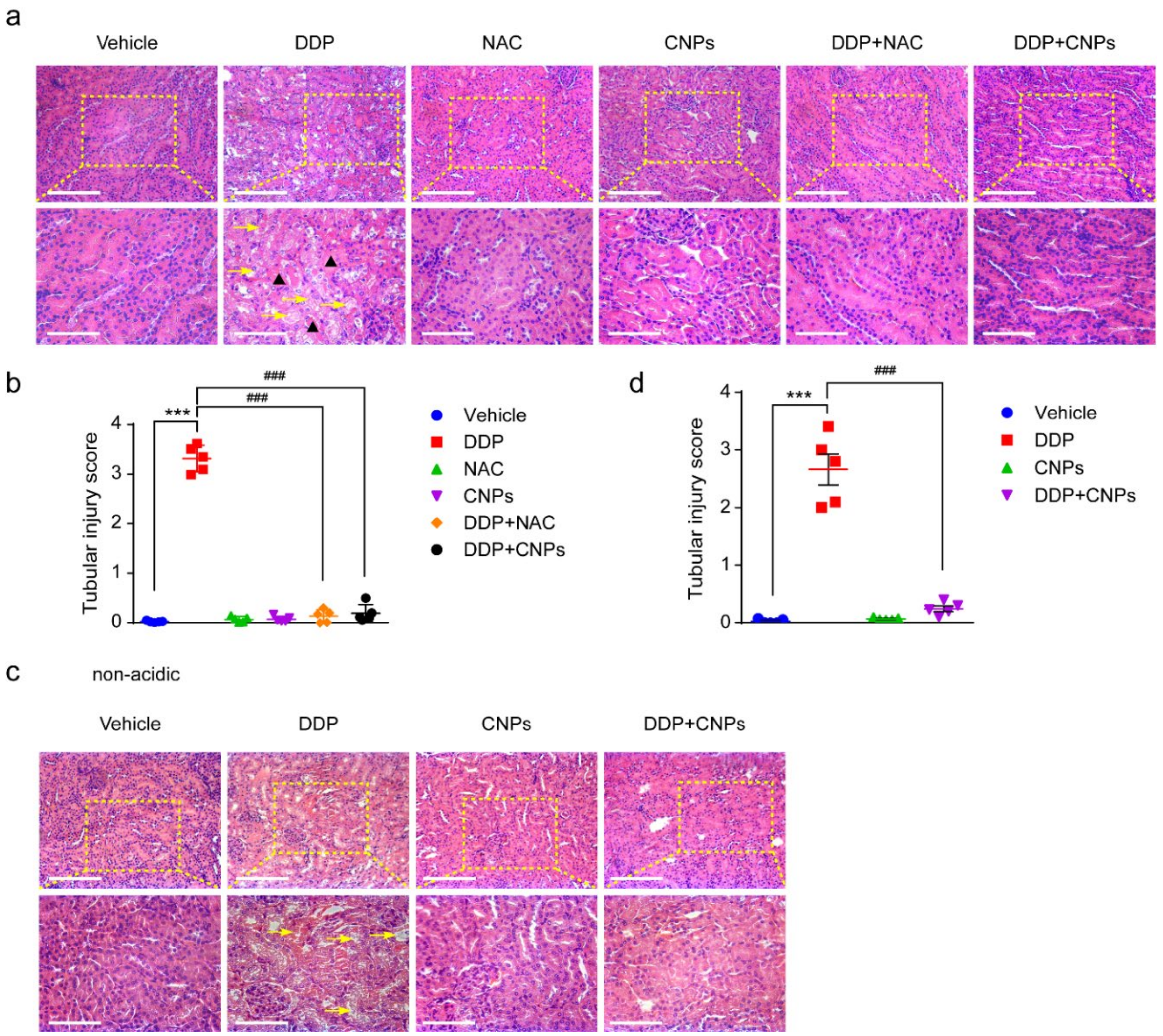

Supplementary Figure 32. CNPs protect against chemotherapy-induced AKI in tumour-bearing mice. a, Representative H&E-stained sections of kidneys in nude mice fed with sterile water after different treatments. n = 5 independent animals/group. **b**, The tubular injury scores of nude mice fed with sterile water after different treatments. n = 5 independent animals, $P_{(DDP)}$ = 1.8E-14, $P_{(DDP+NAC)}$= 1.8E-14, $P_{(DDP+CNPs)}$ = 1.8E-14. **c**, Representative H&E-stained sections of kidneys in nude mice fed with soda water after different treatments. n = 5 independent animals. **d**, The tubular injury scores of nude mice fed with soda water after different treatments. The lower panel are the magnified regions from the upper panel. Scale bar: 200 μm (up) or 100 μm (down). n = 5 independent animals, $P_{(DDP)}$ = 1.8E-09, $P_{(DDP+CNPs)}$ = 6.3E-09. Data are presented as means ± SEM., ****P* < 0.001, ###*P* < 0.001; one-way ANOVA with multiple comparisons test. Source data are provided as a Source Data file.

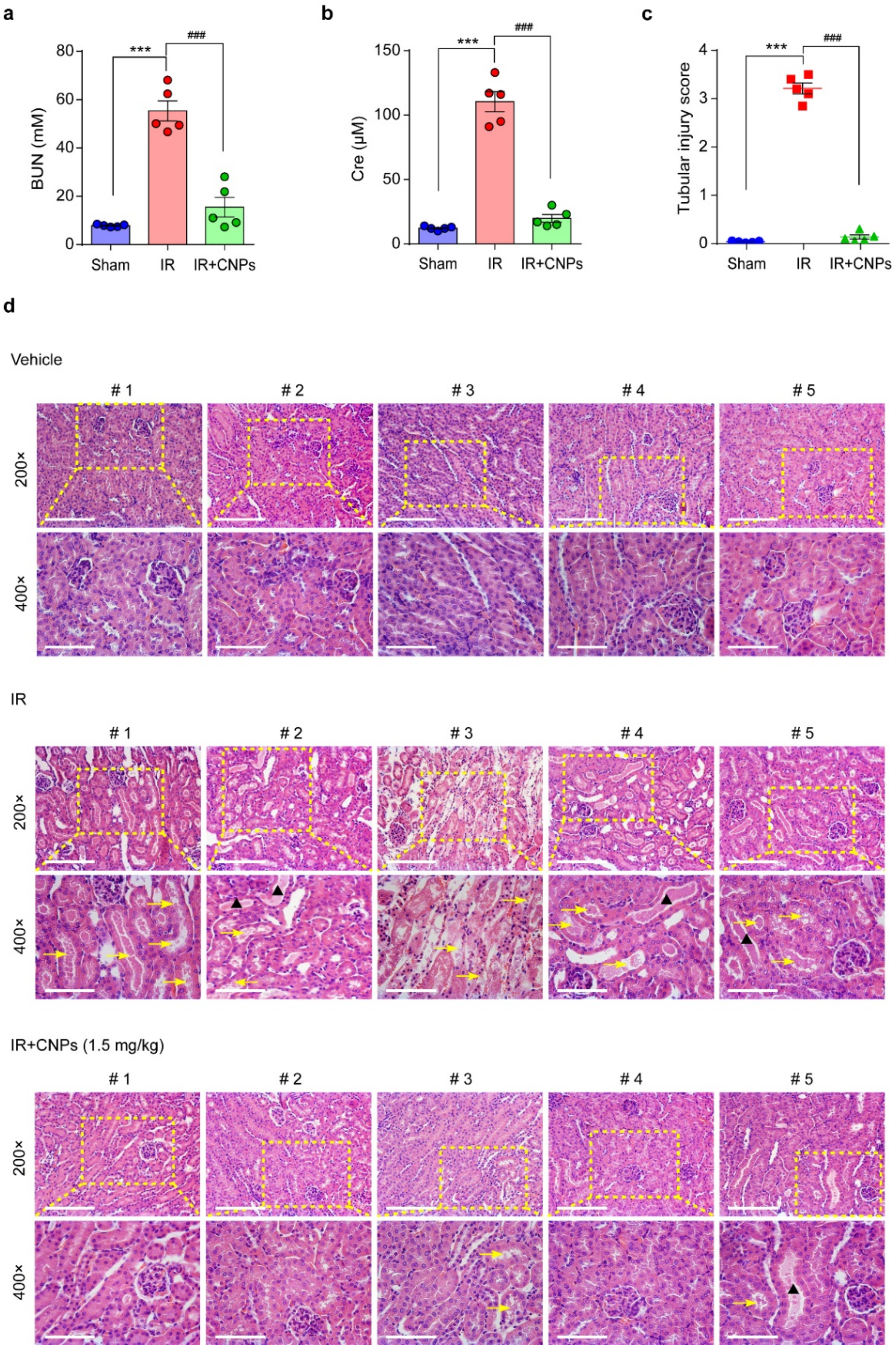

Supplementary Figure 33. CNPs protect ischemia-reperfusion (IR)-induced AKI. a, **b**, Serum BUN (**a**) and Cre (**b**) levels of IR-induced AKI mice after different treatments. n = 5 independent animals. In **a**, $P_{(IR)}$ = 1.01E-06, $P_{(IR+CNPs)}$ = 6.5E-06; in **b**, $P_{(IR)}$ = 1.7E-08, $P_{(IR+CNPs)}$ = 4.2E-08. **c**, The tubular injury score was calculated according to the percentage of damaged tubules as reported: 0, no damage; 1, <25% damage; 2, 25-50% damage; 3, 50-75% damage;

4, >75% damage. A pathologist evaluated 5 randomly selected fields per section of the mouse kidneys at a magnification of ×400 in a blind manner. n = 5 independent animals, $P_{(IR)}$ = 1.27E-12, $P_{(IR+CNPs)}$ = 1.59E-12. **d**, Representative H&E sections of the kidneys from each group. n=5 independent animals. Arrows indicate tubules with necrosis, epithelial anoikis cavitation, or loss of brush border. Triangles denote cast formation in tubes. The lower panel are the magnified regions from the upper panel. Scale bar: 200 μm (up) or 100 μm (down). Data are presented as means ± SEM., ***P < 0.001, ###P < 0.001; one-way ANOVA with multiple comparisons test. Source data are provided as a Source Data file.

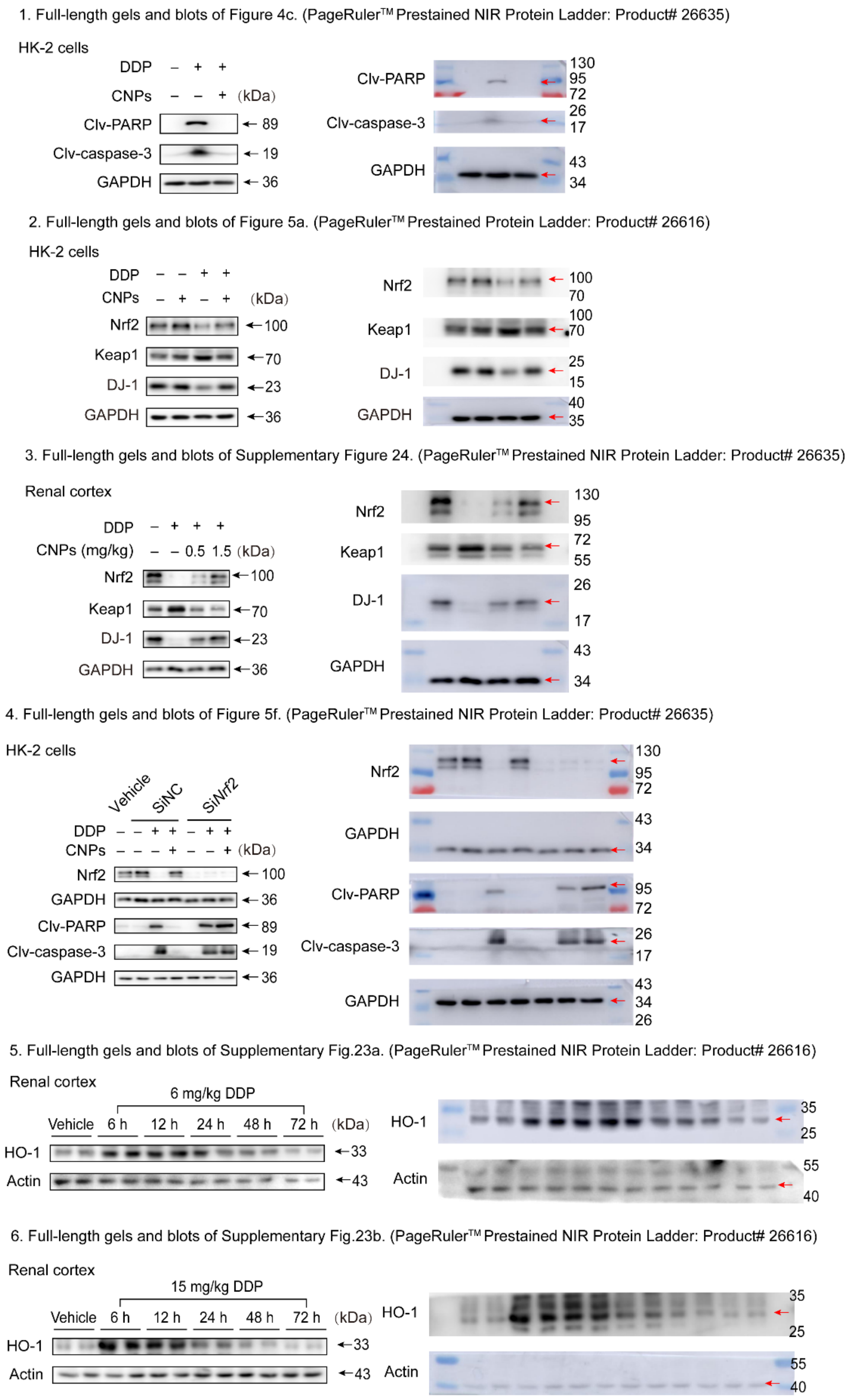

Supplementary Figure 34. The respective original western blot images. The western blot images presented in papers and their corresponding original images.

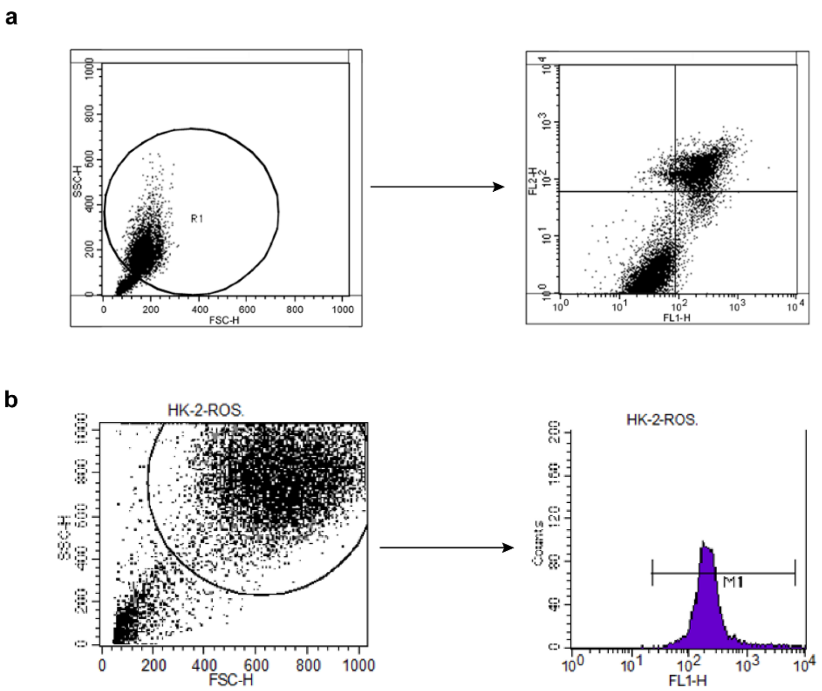

Supplementary Figure 35. Gating strategies of flow cytometry. (**a**) Gating strategy to determine the percentage of Annexin V-FITC^{+}PI^{+} and Annexin V-FITC^{+}-PI^{-} cells presented in Fig. 4a. (**b**) Gating strategy to determine the intracellular ROS level presented in Fig. 4f.

Table S1:

Catalyst	pH	[E] (mg mL^{-1})	Substrate	K_M (M)	Vmax (mg L^{-1} min^{-1})
CNPs	7.4	3.6×10^{-2}	H_2O_2	0.06948	0.43
CNPs	6.6	3.6×10^{-2}	H_2O_2	0.09469	0.21

Table S1. The Michaelis–Menten constant (K_M) and maximum reaction rate(V_{max}) of as-prepared CNPs with H_2O_2 as the substrate for CAT-like catalysis under different pH conditions. Source data are provided as a Source Data file.

Table S2:

Serial number	Primer name	Forward sequences	Reverse sequences
1	Mouse *KIM-1*	5-CAGGAAGACCCACGACTATTTC-3	5-GTGTGTAGATGTTGGAGGAGTG-3
2	Mouse *HO-1*	5-CTCTCTTCTCTTGGGCCTCTAA-3	5-TGTCAGGTATCTCCCTCCATTC-3
3	Human *HO-1*	5-TCTTGGCTGGCTTCCTTAC-3	5-CATAGGCTCCTTCCTCCTTTC-3
4	Mouse *NOX2*	5-GAAAACTCCTTGGGTCAGCACT-3	5-ATTTCGACACACTGGCAGCA-3
5	Human *NOX2*	5-ACCTCAACTGCAGCCTTATC-3	5-ATCCAACAATCTCCTGGTTCTC-3
6	Mouse *Nrf-2*	5-CTCCGTGGAGTCTTCCATTTAC-3	5-GCACTATCTAGCTCCTCCATTTC-3
7	Mouse *Keap1*	5-CTCTGAGCCCTGAACAGTTATT-3	5-CTGTGTGCCTACTCCATTCTT-3
8	Mouse *DJ-1*	5-GCAGTGTAGCCGTGATGTAA-3	5-CACCATAGGCGACTCAGATAAA-3
9	Mouse *GAPDH*	5-TCAACAGCAACTCCCACTCTTCCA-3	5-ACCCTGTTGCTGTAGCCGTATTCA-3
10	Human *GAPDH*	5-GGTGTGAACCATGAGAAGTATGA-3	5-GAGTCCTTCCACGATACCAAAG-3
11	Human Si*Nrf2*	5-GGUUGAGACUACCAUGGUUTT-3	5-AACCAUGGUAGUCUCAACCTT-3

Table S2: Sequences of the primers used for qRT-PCR.
