# Supplementary material for: Catalytic activity tunable ceria nanoparticles prevent chemotherapy-induced acute kidney injury without interference with chemotherapeutics
Source: Nat Commun. 2021 Mar 4;12:1436. doi: 10.1038/s41467-021-21714-2 (PMC7933428; doi:10.1038/s41467-021-21714-2)
Supplement: Supplementary file 3 — Reporting Summary [file 41467_2021_21714_MOESM3_ESM.pdf]

nature research

Corresponding author(s): Daishun Ling

Last updated by author(s): Dec 10, 2020

Reporting Summary

Nature Research wishes to improve the reproducibility of the work that we publish. This form provides structure for consistency and transparency in reporting. For further information on Nature Research policies, see our Editorial Policies and the Editorial Policy Checklist.

Statistics

For all statistical analyses, confirm that the following items are present in the figure legend, table legend, main text, or Methods section.

n/a	Confirmed	
☐	☒	The exact sample size (n) for each experimental group/condition, given as a discrete number and unit of measurement
☐	☒	A statement on whether measurements were taken from distinct samples or whether the same sample was measured repeatedly
☐	☒	The statistical test(s) used AND whether they are one- or two-sided<br>*Only common tests should be described solely by name; describe more complex techniques in the Methods section.*
☒	☐	A description of all covariates tested
☒	☐	A description of any assumptions or corrections, such as tests of normality and adjustment for multiple comparisons
☐	☒	A full description of the statistical parameters including central tendency (e.g. means) or other basic estimates (e.g. regression coefficient) AND variation (e.g. standard deviation) or associated estimates of uncertainty (e.g. confidence intervals)
☐	☒	For null hypothesis testing, the test statistic (e.g. F, t, r) with confidence intervals, effect sizes, degrees of freedom and P value noted<br>*Give P values as exact values whenever suitable.*
☒	☐	For Bayesian analysis, information on the choice of priors and Markov chain Monte Carlo settings
☒	☐	For hierarchical and complex designs, identification of the appropriate level for tests and full reporting of outcomes
☒	☐	Estimates of effect sizes (e.g. Cohen's d, Pearson's r), indicating how they were calculated

Our web collection on statistics for biologists contains articles on many of the points above.

Software and code

Policy information about availability of computer code

Data collection	The catalytic activities was recorded using a specific oxygen electrode on Multi-Parameter Analyzer (Leici, China) or a Total Superoxide Dismutase Assay Kit ((Beyotime, China). The quantification of ceria nanoparticles in plasma and tissues was analyzed by using ICP-MS (Agilent Technologies 7800). Western blot was carried out using enhanced chemiluminescence (ECL) detection reagents (FDbio Science Biotech Co., Ltd., Hangzhou, China). qRT-PCR was carried out using SYBR Green Supermix (Bio-Rad, Hercules, California, USA). Flow cytometry was performed using fluorescence microscope (Leica, DMI3000B, Germany).
Data analysis	Statistical comparisons were performed using Microsoft Excel (Version 1808), Graphpad Prism (version 8.0) and Image J (version 1.8.0)

For manuscripts utilizing custom algorithms or software that are central to the research but not yet described in published literature, software must be made available to editors and reviewers. We strongly encourage code deposition in a community repository (e.g. GitHub). See the Nature Research guidelines for submitting code & software for further information.

Data

Policy information about availability of data

All manuscripts must include a data availability statement. This statement should provide the following information, where applicable:

- Accession codes, unique identifiers, or web links for publicly available datasets
- A list of figures that have associated raw data
- A description of any restrictions on data availability

The authors declare that the main data supporting the findings of this study are available in this Article, its Supplementary Information and Source Data. Extra data for the individual measurements are available on reasonable request. The source data underlying Figs. 1c–e, 2a–g, 3b-e, 4b-c, e, g-k, 5a–d, f-h, 6b–c, e-f, h-j as well as Supplementary Figs. 1-7, 8a-h, 9, 10a, b, 11, 13-15, 18b, 19b, c, 20b, 21, 22a-d, 24-27, 28a, b, 29a, b, 30a, b, 31a, b, 32b, d, 33a-c are provided as a Source Data file.

Field-specific reporting

Please select the one below that is the best fit for your research. If you are not sure, read the appropriate sections before making your selection.

- [x] Life sciences
- [ ] Behavioural & social sciences
- [ ] Ecological, evolutionary & environmental sciences

For a reference copy of the document with all sections, see nature.com/documents/nr-reporting-summary-flat.pdf

Life sciences study design

All studies must disclose on these points even when the disclosure is negative.

Sample size	Sample size choice was based on previous studies (ref. He, X., Zhang, L., Queme, L. et al, 2018. https://doi.org/10.1038/nm.4483; Huang, J., Li, J., Lyu, Y. et al. https://doi.org/10.1038/s41563-019-0378-4; Ni, D., Jiang, D., Kutyreff, C.J. et al. Ni, D., Jiang, D., Kutyreff, C.J. et al. ; Jiang, D., Ge, Z., Im, HJ. et al. https://doi.org/10.1038/s41551-018-0317-8; Deng, H., Yang, W., Zhou, Z. et al. https://doi.org/10.1038/s41467-020-18745-6), not predetermined by a statistical method. Sample sizes were indicated in the legend of each Figure and Supplementary Figure.
Data exclusions	No data were excluded.
Replication	We confirm all attempts at replication were successful. The experiments of western blots were independently replicated at least twice, and other experimental findings were all replicated at least 3 times.
Randomization	All samples were randomly allocated into experimental groups.
Blinding	Investigators were not blinded for nanomaterial synthesis. For in vivo experiments, the investigators were blinded to group allocation during data collection and analysis. However, The pharmacokinetic study was performed unblinded and all subsequent steps of data analysis were also unblinded because determination of nanoparticles concentrations and PK parameters are considered as objective measures, not subject to bias.

Reporting for specific materials, systems and methods

We require information from authors about some types of materials, experimental systems and methods used in many studies. Here, indicate whether each material, system or method listed is relevant to your study. If you are not sure if a list item applies to your research, read the appropriate section before selecting a response.

Materials & experimental systems

n/a	Involved in the study	
[ ]	[x]	Antibodies
[ ]	[x]	Eukaryotic cell lines
[x]	[ ]	Palaeontology and archaeology
[ ]	[x]	Animals and other organisms
[x]	[ ]	Human research participants
[x]	[ ]	Clinical data
[x]	[ ]	Dual use research of concern

Methods

n/a	Involved in the study	
[x]	[ ]	ChIP-seq
[ ]	[x]	Flow cytometry
[x]	[ ]	MRI-based neuroimaging

Antibodies

Antibodies used	anti-Nrf2 (1:1000, ab62352), Abcam: Anti-Nrf2 antibody [EP1808Y], Cat number: ab62352, Clone name: Rabbit monoclonal, Lot Number: GR3231661-4; anti-Keap1(1:1000, ab119403), Abcam: Anti-Keap1 antibody [1B4], Cat number: ab119403, Clone name: Mouse monoclonal, Lot Number: GR3325770-1; anti-HO-1(1:1000, ab68477), Abcam: Anti-Heme Oxygenase 1 antibody [EPR1390Y] Cat number: ab68477, Clone name: Rabbit monoclonal, Lot Number: GR325245-14; anti-cleaved caspase-3 (1:1000, ab49822), Abcam: Anti-Cleaved Caspase-3 antibody, Cat number: ab49822, Clone name: Rabbit polyclonal, Lot Number: GR3191900-12; anti-cleaved PARP (1:1000, ET1608-10, human), Huabio: Anti-Cleaved PARP antibody [SU0314], Cat number: ET1608-10, Clone name: Recombinant Rabbit monoclonal IgG, Lot Number: HL1224; anti-cleaved PARP (1:1000, 9544s, mouse) , Cell Signaling Technology: Cleaved PARP (Asp214) Antibody (Mouse Specific), Cat number: 9544s, Clone name: Rabbit, Lot Number: 5; anti-DJ-1(1:1000, sc-55572), Santa Cruz Biotechnology: DJ-1 Antibody (D-4), Cat number: sc-55572, Clone name: Mouse monoclonal, Lot number: K2014; anti-Actin (1:1000, sc-1615), Santa Cruz Biotechnology: Actin Antibody (C-11), Cat number: sc-1615, Clone name: goat polyclonal, Lot number: I1415; anti-GAPDH (1:1000, db106), DiagBio technology: Anti-GAPDH antibody, Clone name: Rabbit Polyclonal, Lot number: P1020, Species Reactivity: Human, Mouse, Rat; 1. Clone name: Goat Polyclonal, HANGZHOU FUDE BIOLOGICAL TECHNOLOGY: Goat Anti-Mouse HRP, Cat number: FDM007.

Validation	Rabbit anti-Nrf2 antibody<br>Suitable for: Flow Cyt, ChIP, ICC/IF, WB, IP, IHC-P<br>https://www.abcam.com/nrf2-antibody-ep1808y-chip-grade-ab62352.html<br><br>Mouse anti-Keap1 antibody<br>Suitable for: ICC/IF, WB, IHC-P, Flow Cyt<br>https://www.abcam.com/keap1-antibody-1b4-ab119403.html<br><br>Rabbit anti-HO-1 antibody<br>Suitable for: ICC/IF, WB, IP, Flow Cyt<br>https://www.abcam.com/heme-oxygenase-1-antibody-epr1390y-ab68477.html<br><br>Rabbit anti-cleaved caspase-3 antibody<br>Suitable for: WB, ICC/IF<br>https://www.abcam.com/cleaved-caspase-3-antibody-ab49822.html<br><br>Rabbit anti-cleaved PARP antibody<br>Applications: WB, ICC, IP, FC<br>http://www.huabio.cn/search?sort=asc&keyword=ET1608-10<br><br>Rabbit anti-cleaved PARP antibody<br>Application: Western Blotting<br>https://www.cellsignal.com/products/primary-antibodies/cleaved-parp-asp214-antibody-mouse-specific/9544?site-search-type=Products&N=4294956287&Ntt=9544s&fromPage=plp&_requestid=4259222<br><br>Mouse anti-DJ-1 antibody<br>Application: Western Blotting, immunoprecipitation, immunofluorescence, immunohistochemistry and solid phase ELISA<br>https://www.scbt.com/p/dj-1-antibody-d-4?requestFrom=search<br><br>Goat anti-Actin antibody<br>Application: Western Blotting<br>https://www.scbt.com/p/actin-antibody-c-11?requestFrom=search<br><br>Rabbit anti-GAPDH antibody<br>Applications: WB, IHC, ICC/IF, FC, IP<br>http://www.diagbio.com/prodetail.aspx?caid3=73&pid=31405

Eukaryotic cell lines

Policy information about cell lines

Cell line source(s)	HK-2, ES-2, OVCAR8, A549 cells, HepG2 cells and A549 cells were purchased from American Type Culture Collection (ATCC, Manassas, VA). L02 cells were purchased from Shanghai Institute of Biochemistry and Cell Biology (Shanghai, China).
Authentication	Cells were used without modification once received from the supplier and therefore were not authenticated.
Mycoplasma contamination	The cell line was tested negative for Mycoplasm contamination per suppliers.
Commonly misidentified lines (See ICLAC register)	No commonly misidentified lines were used.

Animals and other organisms

Policy information about studies involving animals; ARRIVE guidelines recommended for reporting animal research

Laboratory animals	ICR mice (Male and female, 6-8 weeks, Beijing Vital River Laboratory Animal Technology Co., Ltd.) were used in in vivo experiment for assessment of AKI treatment.<br>BALB/c nude mice (Female, 6-8 weeks, Shanghai SLAC Laboratory Animal Co., Ltd.) were used in in vivo experiment about tumour treatment and AKI assessment.
Wild animals	Wild animals were not involved in this study.

Field-collected samples	Field-collected samples were not involved in this study.
Ethics oversight	The use and care of the mice were in accordance with the guidelines of the Institutional Animal Care and Use Committee (IACUC) of Zhejiang University. All procedures were approved by the IACUC of Zhejiang University.

Note that full information on the approval of the study protocol must also be provided in the manuscript.

Flow Cytometry

Plots

Confirm that:

- [x] The axis labels state the marker and fluorochrome used (e.g. CD4-FITC).
- [x] The axis scales are clearly visible. Include numbers along axes only for bottom left plot of group (a 'group' is an analysis of identical markers).
- [x] All plots are contour plots with outliers or pseudocolor plots.
- [x] A numerical value for number of cells or percentage (with statistics) is provided.

Methodology

Sample preparation	Flow cytometry analysis of cell apoptosis: HK-2 cells were cultured in 6-well plates (2 × 105 per well) for 24 h. Afterwards, the culture mediums were respectively treated with vehicle (DMSO), DDP (10 μM) as well as DDP and CNPs (50 μM), and incubated for another 24 h. Cells were collected and incubated with a FITC Annexin V Apoptosis Detection Kit (BD PharmingenTM, USA) for 15 min at room temperature, an then resuspended with 400 μL 1×binding buffer. The intensity of fluorescence was measured by flow cytometer (BD Biosciences, FACSuiteTM, USA) within 1 h.<br>Cellular ROS detection: HK-2 cells were cultured in 6-well confocal plates (2×105 cells per well). The cells were treated with 10 μM cisplatin and 50 μM CNPs, then co-incubated for 24 h. Cells were collected and incubated with Fluorescence probe (2,7-dichlorodihydrofluorescein diacetate) for 30 min. Then cells were washed by 1XPBS for 3 times and then resuspended. ROS were detected by the FITC channel for fluorescence intensity using flow cytometry (BD Biosciences, FACSuiteTM, USA).
Instrument	BD FACSVERS
Software	BD FACSVERS
Cell population abundance	By stained with specific fluoresence-labeled antibodies, cells were separated into different parts and cytometry can calculate the normal or apoptosis fractions because of their damaged conditions.
Gating strategy	For cell apoptosis detection, Annexin V-FITC was used to label apoptotic and necrotic cells, and PI was used to label necrotic cells. PBS-treated cells were stained with/without Annexin-V-FITC or PI to determine gate. For intracellular ROS level detection, 1×PBS-treated cells were stained without DCFH-DA to determine an appropriate gate.

- [x] Tick this box to confirm that a figure exemplifying the gating strategy is provided in the Supplementary Information.
